# Memory retrieval, reconsolidation, and extinction: Exploring the boundary conditions of post-conditioning cue exposure

**DOI:** 10.3389/fnsyn.2023.1146665

**Published:** 2023-03-02

**Authors:** Nicole C. Ferrara, Janine L. Kwapis, Sydney Trask

**Affiliations:** ^1^Discipline of Physiology and Biophysics, Rosalind Franklin University, North Chicago, IL, United States; ^2^Center for Neurobiology of Stress Resilience and Psychiatric Disorders, Rosalind Franklin University, North Chicago, IL, United States; ^3^Department of Biology, The Pennsylvania State University, University Park, PA, United States; ^4^Department of Psychological Sciences, Purdue University, West Lafayette, IN, United States; ^5^Purdue Institute for Integrative Neuroscience, West Lafayette, IN, United States

**Keywords:** fear, extinction, reconsolidation, memory, amygdala

## Abstract

Following fear conditioning, behavior can be reduced by giving many CS-alone presentations in a process known as extinction or by presenting a few CS-alone presentations and interfering with subsequent memory reconsolidation. While the two share procedural similarities, both the behavioral outcomes and the neurobiological underpinnings are distinct. Here we review the neural and behavioral mechanisms that produce these separate behavioral reductions, as well as some factors that determine whether or not a retrieval-dependent reconsolidation process or an extinction process will be in effect.

## Introduction

Almost a century has passed since Pavlov’s (1927) initial demonstration of extinction, a phenomenon in which conditional responding declines when a conditional stimulus (CS) is no longer presented with a biologically relevant unconditional stimulus (UCS). While significant strides have been made in understanding the behavioral and neurobiological mechanisms of Pavlovian extinction learning, it remains an active area of research due to findings that demonstrate extinguished responding will readily return under a variety of circumstances. These “relapse” effects (including spontaneous recovery, renewal, and reinstatement) suggest behavioral changes produced by extinction are impermanent and CS-UCS learning remains intact throughout extinction. While a variety of strategies have been employed to reduce return of extinguished responding (Brooks and Bouton, 1993, 1994; Vansteenwegen et al., 2006; Culver et al., 2011), behavioral relapse is the rule rather than the exception (Bouton et al., 2021). This presents a significant hurdle to extinction-based exposure therapies aiming to reduce maladaptive responding stemming from classically conditioned experiences (Bouton et al., 2001; Hermans et al., 2006).

More recently, researchers have aimed to circumvent issues associated with extinction by directly targeting the original CS-UCS memory, taking advantage of a brief period of memory lability following retrieval known as reconsolidation. During this post-retrieval period, physical (Misanin et al., 1968) or pharmacological (Nader et al., 2000) assaults can fundamentally change the memory. Reconsolidation is thought to alter or ablate the original memory trace itself, particularly within regions like the amygdala, hippocampus, and prefrontal cortex. As such, memory that has been reduced through reconsolidation is not susceptible to relapse.

However, there are procedural similarities between retrieval-induced reconsolidation and extinction: both involve some amount of exposure to unreinforced conditional stimuli. A key difference between the two is that reconsolidation typically involves limited or brief exposure to a CS whereas extinction involves more extensive exposure to the CS. Further, much of the mechanistic work has suggested that extinction-related and reconsolidation-related fear inhibition depend differentially on amygdala interactions with the hippocampus and prefrontal cortex.

Understanding the conditions that produce extinction or reconsolidation-like effects will aid the development of therapeutic strategies that aim to take advantage of reconsolidation to limit behavioral relapse associated with extinction learning. Here, we review the literature underlying the boundaries between what variables produce retrieval-induced reconsolidation and which variables result in the creation of a new memory via extinction. We will discuss both the behavioral and neural mechanisms (see Table 1), with a primary focus on basolateral amygdala (BLA), dorsal hippocampal (DH), and infralimbic prefrontal cortical (IL) contributions.

**TABLE 1:** Brief overview summarizing similarities and differences between extinction and reconsolidation.

Finding	Extinction	Reconsolidation
Susceptible to spontaneous recovery	Yes	No
Susceptible to renewal	Yes	No
Susceptible to reinstatement	Yes	No
Observed after many trials	Yes	No
Observed after few trials	No	Yes
Need prediction error	Yes	Yes
Produces changes in synaptic GluA2 in BLA	No	Yes
Requires protein synthesis	Yes	Yes

While these represent the most commonly observed findings, some exceptions exist; see text for details.

## Fear memory formation

Fear memories are formed through CS-UCS pairings, resulting in an association between the two supported by an amygdala-centric neural circuit (Helmstetter et al., 2008). Following fear conditioning, memory strength can be indicated by the amount of fear responding. While several brain regions are critical for fear processes, medial prefrontal cortical, DH, and BLA subregions are especially critical for memory formation and retention (Kwapis et al., 2011; Gilmartin et al., 2012; Likhtik et al., 2014; Zelikowsky et al., 2014). The interaction between these regions governs the amount of fear and subsequent memory strength, precision, and the ability to later modify memory.

Fear memory is supported by changes in synaptic strength and plasticity in the BLA, characterized by AMPA receptor synaptic presence and NMDA-dependent long-term potentiation (Clugnet and LeDoux, 1990; Maren and Fanselow, 1995; Rogan and LeDoux, 1995; Humeau et al., 2007). Synaptic potentiation is supported by several intracellular processes that contribute to changes in gene expression and the synthesis of new proteins, including MAPK and PKA cellular signaling, epigenetic modifications, and subsequent changes in gene expression. These together support persistent changes needed for long-term memory storage. Fear memory formation can be disrupted by BLA inactivation with inhibitory GABAergic manipulations (Helmstetter and Bellgowan, 1994; Gilmartin et al., 2012), interference with cellular signaling cascades (Schafe and LeDoux, 2000; Schafe et al., 2000; Parsons et al., 2006), blockade of epigenetic modifiers (Monsey et al., 2011; Maddox et al., 2013a,b), or disruptions in glutamatergic receptor trafficking (Joels and Lamprecht, 2010; Migues et al., 2010).

## Extinction learning and inhibitory memory creation

Following memory formation, behavior can be reduced through extinction, where a CS or context that was previously predictive of a UCS is presented alone. While retrieval-based reconsolidation is observed after relatively few CS-alone presentations, extinction is observed following many CS-alone presentations. Extinction results in the formation of a new, context-dependent CS-No UCS memory (Bouton and Bolles, 1979; Trask et al., 2017).

Extinction is dependent on BLA inhibitory GABAergic neuronal activity (Akirav et al., 2006; Sangha et al., 2009; Heldt et al., 2012). Synaptic plasticity within the BLA is governed by NMDA receptor activity, which triggers cellular and synaptic changes needed to support long-term memory. Because extinction learning represents a new form of learning, it requires many of the same modifications as initial memory formation (Walker et al., 2002; Zimmerman and Maren, 2010). While these effects are often thought to occur on glutamatergic neurons, GABAergic populations also contain NMDA receptors and GABAergic neuronal activity is mediated by NMDA receptors (Royer and Pare, 2002; Szinyei et al., 2003; Polepalli et al., 2010; Spampanato et al., 2011). Thus, NMDA receptor effects may result from GABAergic regulation.

The increase in GABAergic tone within the BLA decreases the activity of excitatory neurons needed for fear expression. Extinction learning is dependent on IL projections to the BLA; direct IL inputs to the BLA engage GABAergic neurons that suppress principal excitatory neurons (Selleck et al., 2018; Bukalo et al., 2021). Extinction increases the excitability of BLA-projecting IL neurons to increase the capacity of the IL to suppress BLA activity (Bloodgood et al., 2018; Ferrara et al., 2020). Inhibiting IL activity during extinction learning impairs its acquisition (Sierra-Mercado et al., 2011; Do-Monte et al., 2015) and epigenetic manipulations within the IL modify the strength and persistence of extinction memories (Lattal et al., 2007; Marek et al., 2011; Bahari-Javan et al., 2012; Stafford et al., 2012; Kwapis and Wood, 2014), suggesting plasticity in this region is critical for extinction. Similarly, manipulations that directly suppress BLA excitation enhance extinction. This includes both manipulations that activate GABAergic inputs from the mPFC, and those that inhibit excitatory inputs to the BLA from the auditory thalamus and cortex (Cho et al., 2013; Ferrara et al., 2020). In this manner, extinction memories are thought to recruit BLA inhibition to suppress the original, excitatory fear memory. However, several results have demonstrated that IL activity is not necessary for the expression of extinguished behavior (Do-Monte et al., 2015), which instead depends on the ventral hippocampus (Sierra-Mercado et al., 2011), likely through projections inhibiting the prelimbic cortex (Vasquez et al., 2019).

Extinguished fear renews when tested outside of the extinction context or after a sufficient amount of time (Bouton et al., 2006). The DH is important for encoding contextual attributes of a memory, and DH lesions prevent context-dependent return of fear after extinction (Corcoran and Maren, 2004; Ji and Maren, 2005; Helmstetter et al., 2008; Gafford et al., 2011). However, DH-dependency is based on the context in which renewal occurs, as DH regulates renewal in novel contexts but not the training context (Corcoran and Maren, 2004). Impaired extinction is believed to be a result of increased BLA-DH synchronization when GABAergic processes are disrupted (Sangha et al., 2009). Importantly, changes at inputs supporting initial fear memory storage remain unchanged after fear extinction, suggesting that the return of fear following extinction is a result of a persisting neural trace (Kim and Cho, 2017).

Together, this suggests extinction memory is characterized by increases in GABAergic tone, dependent on IL-BLA pathway, and persisting/relapsed fear is supported by DH-BLA interaction. Activity among these regions drives fear expression following extinction.

## Retrieval-induced memory lability and reconsolidation

Following retrieval, memory becomes temporarily sensitive to disruption and must be stored again in a process called reconsolidation. In an initial demonstration, following CS retrieval, rats were given electroconvulsive shock and substantially decreased their CS-elicited fear relative to controls that did not receive electroconvulsive shock (Misanin et al., 1968). Other types of amnesic events following retrieval, like hypothermia, have similar deleterious effects on memory (Mactutus et al., 1979). Interest in this topic renewed following work that demonstrated protein synthesis blockade with BLA anisomycin infusions immediately after cued fear retrieval impaired later CS memory (Nader et al., 2000) and conditions that create this effect began to be explored.

Early experiments compared how post-retrieval and extinction injections of anisomycin affected behavior. BLA protein synthesis inhibition following either retrieval or extinction resulted in impaired reconsolidation, consistent with a weaker fear memory (Duvarci et al., 2006). One likely explanation for this finding is that extinction and reconsolidation rely on non-overlapping circuitry, with extinction processes being dependent on input from the infralimbic cortex (e.g., Do-Monte et al., 2015). Interestingly, post-retrieval anisomycin prevented both spontaneous recovery and reinstatement, suggesting that retrieval-based manipulations may affect the original memory itself (Duvarci and Nader, 2004). The efficacy of anisomycin inhibition in the BLA is contingent on brief decreases in AMPA receptor-mediated synaptic transmission. Memory retrieval is characterized by persistent increases in AMPA- relative to NMDA-mediated currents, with transient changes in the contribution of calcium-impermeable and calcium-permeable AMPA receptors (Hong et al., 2013). While calcium-impermeable AMPA receptors are present in BLA synapses, these AMPA receptors internalize and are replaced with calcium-permeable AMPA receptors (Hong et al., 2013). This exchange is believed to mediate intracellular plastic changes required for memory lability, as this AMPA receptor trafficking occurs along the same timeframe that anisomycin impairs memory retention and interferes with anisomycin memory impairments when this transient internalization of calcium impermeable AMPA receptors is inhibited (Hong et al., 2013). This work identified the importance of AMPA receptor trafficking, and specifically calcium influx for protein synthesis-dependent memory lability required for memory modification.

A number of changes occur within the BLA during fear memory reconsolidation, including intracellular signaling cascades, epigenetic modifications, and changes in gene expression (Johansen et al., 2011; Kwapis and Wood, 2014). One particularly important intracellular change that occurs in the BLA is altered protein degradation, critical for memory destabilization (Jarome et al., 2011, 2013, 2016). Transient internalization of calcium-impermeable AMPA receptors allows for postsynaptic calcium influx, and this regulates protein degradation-related activity (Ferrara et al., 2019a). Some of the proteins targeted for degradation include synaptic scaffolds and factors mediating protein synthesis, suggesting the proteasome system plays an important role in the reorganization of synaptic structure following memory retrieval and in the regulation of anisomycin-related memory impairments (Jarome et al., 2011). Interestingly, these processes are regulated by CaMKII activity, which is initiated by calcium influx (Jarome et al., 2013, 2016). Inhibition of any of these factors prevents protein synthesis and subsequent effects of anisomycin. Cellular mechanisms downstream from AMPA receptor trafficking therefore play a pivotal role in preservation of fear memory.

The necessity of protein synthesis was also extended to hippocampus-dependent context fear memories, where local DH anisomycin infusions reduced fear (Debiec et al., 2002). In a delay fear conditioning task new contextual information introduced during memory retrieval is sufficient to drive reconsolidation processes and the absence of new contextual information leaves memory resistant to disruption (Jarome et al., 2015). Synaptic activity in the BLA following memory retrieval in a new context is mediated by DH activity; inhibition of DH activity reduces the necessity of contextual novelty for reconsolidation-induced changes in behavior and calcium-impermeable GluA2-containing AMPA receptor trafficking within the synapse (Ferrara et al., 2019b). Together, these results suggest that the DH and BLA are critical sites for fear memory storage and can influence reconsolidation-like processes.

Some work has demonstrated that prediction error is necessary to induce reconsolidation-like effects (Sevenster et al., 2014), in line with findings suggesting new information must be included in to induce memory lability during retrieval (see Exton-McGuinness et al., 2015, for a review). However, prediction error may not be the only factor driving memory lability accompanied by a brief retrieval. This has been clearly seen when a CS-UCS *retrieval* presentation (i.e., no prediction error is likely as the CS is paired with what is predicted from initial learning) still leaves a memory susceptible to disruption with a post-retrieval anisomycin injection (Duvarci and Nader, 2004). Interestingly, this is only the case in situations in which the memory is not well-learned, suggesting the ability for protein synthesis inhibition to impair a memory without the introduction of prediction error may instead be reflective of impaired consolidation that accompanies new learning rather than impaired reconsolidation indicative of retrieval of a well-learned memory.

New information integrated during memory retrieval and mediated by reconsolidation can also change the neural circuitry that supports memory, suggesting that reorganization happens more broadly than at the synapse. Memory updating with a single trial of delay conditioning following initial trace fear conditioning, which is dependent on cortical structures, resulted in responding that was no longer dependent on cortex for expression (Kwapis et al., 2017). This suggests that new information integrated during the single-trial updating session can not only change behavior but also can change the neural circuit that supports fear memory. The reorganized circuit supporting updated memory is less complex than the circuit that supported the original memory, which might be easier to target therapeutically.

Some work challenges the notion that interfering with reconsolidation in the post-retrieval period impacts the original memory. For example, anisomycin injections following context fear retrieval impaired fear expression the next day but had no impact when testing occurred 21 days later (Lattal and Abel, 2004), in line with early results reporting that reactivated memory impaired by hypothermia-induced amnesia was susceptible to spontaneous recovery but new memory was not (Mactutus et al., 1979). Further, protein synthesis inhibition following fear acquisition has a larger, more persistent effect on behavior than following memory retrieval (Stafford and Lattal, 2009), suggesting that a reactivated memory is not as susceptible to disruption as was originally assumed. These results complement early work on reconsolidation-like effects, in which a second exposure to an amnesic event would rescue memory impairments observed following the first exposure (Hinderliter et al., 1975).

## Interactions between reconsolidation and extinction

Some work has combined retrieval with extinction to enhance extinction learning. For example, Monfils et al. (2009) found that a brief retrieval to open the reconsolidation window followed by an extended extinction session resulted in more effective extinction and reduced behavioral relapse. This suggests reactivation makes the original memory susceptible to disruption and under these conditions, extinction can act on the original memory instead of creating a new memory. This was supported by work showing this effect was dependent on changes in calcium-permeable AMPA receptors in the BLA, while regular extinction was not (Clem and Huganir, 2010). This reactivation-extinction method is especially attractive as a therapeutic target because it is readily applicable to and effective in humans and does not require pharmacological manipulations (Schiller et al., 2010). However, failures to replicate in both humans (Chalkia et al., 2020a) and rats (Luyten and Beckers, 2017) or to verify the Schiller et al. (2010) report (Chalkia et al., 2020b) have caused scrutiny on the generality of these effects.

Additional work has found that unpaired presentations of the UCS during extinction can reduce both fear renewal and rapid reacquisition (Lipp et al., 2021), suggesting that UCS exposures during extinction might engage different processes than CS-alone presentations and capitalize on reconsolidation-like mechanisms (see also Siegel et al., 2022).

## The tipping point: When does retrieval become extinction?

In auditory fear conditioning, a few unreinforced CS presentations following conditioning will result in memory lability (reconsolidation), but many unreinforced CS presentations will result in new inhibitory learning (extinction). Presenting few (4) CSs will engage reconsolidation-like processes (indicated by internalization of GluA2-containing AMPA receptors) but presenting many (40) CSs will result in extinction learning (indicated by decreased CREB phosphorylation). However, inhibiting excitatory thalamic projections in the BLA during a brief retrieval can shift both the behavioral and molecular profile to that of extinction, driving decreased CREB phosphorylation and context-dependent reduction in fear susceptible to fear renewal (Ferrara et al., 2021). It has also been suggested that while prediction error is needed to drive memory reconsolidation (Sevenster et al., 2014), too much prediction error or dissimilarity between memory acquisition and retrieval might instead drive new memory formation like that observed in extinction learning (Sevenster et al., 2013). The specific behavioral protocol that is used may ultimately determine whether reconsolidation or extinction is triggered, depending on whether mPFC inputs to the BLA (and subsequent activation of BLA GABAergic circuitry) is recruited. In this circumstance, brief retrieval sessions engage excitatory BLA processes that are initiated with AMPA receptor trafficking. As the retrieval session is extended, this increasingly recruits the mPFC→BLA pathway increasing BLA GABAergic tone.

Like retrieval and extinction procedures, presenting few or many weak shocks following fear conditioning can differentially impact behavior. While 2 weak shocks following contextual fear conditioning can increase fear elicited by that context, 10 weak shocks decreases contextual fear (Ferrara et al., 2019a). Follow-up work comparing this procedure to extinction found that, unlike extinction, the effects of weak shock transcend context and rely on long-term changes at BLA synapses (Bonanno et al., 2023). However, as with standard retrieval and extinction protocols, the specific conditions that increase freezing behavior (Ferrara et al., 2019a) or weaken freezing behavior (Bonanno et al., 2023), are not yet known; it is unclear how many weak shocks are needed to tip memory from strengthening to weakening. However, fear following weak shock exposures decreases with amount of weak UCS presentations, as reduced-intensity UCS presentations did not need to occur within a single session but could occur over several days (Popik et al., 2020). While it seems to function similarly to CS-alone presentations, work needs to identify any boundary conditions that may exist for this procedure.

## Conclusion

While systematic work directly comparing retrieval-dependent reconsolidation to extinction is still sparse, it seems that number of CS presentations is the crucial variable that drives the behavioral and neurobiological differences between reconsolidation and extinction. Other factors, however, may contribute substantially to these effects. This includes introducing new information (e.g., contextual novelty) and optimizing prediction error, which may contribute to the likelihood that reconsolidation-like processes are preferentially engaged.

## Author contributions

ST, JLK, and NCF wrote the manuscript. All authors contributed to the article and approved the submitted version.
